# Apolipoprotein L1 risk variants associate with prevalent atherosclerotic disease in African American systemic lupus erythematosus patients

**DOI:** 10.1371/journal.pone.0182483

**Published:** 2017-08-29

**Authors:** Ashira Blazer, Binhuan Wang, Danny Simpson, Tomas Kirchhoff, Sean Heffron, Robert M. Clancy, Adriana Heguy, Karina Ray, Matija Snuderl, Jill P. Buyon

**Affiliations:** 1 Department of Medicine, Division of Rheumatology, New York University School of Medicine, New York, New York, United States of America; 2 Department of Population Health, Division of Biostatistics, New York University School of Medicine, New York, New York, United States of America; 3 Department of Population Health, Division of Genomics, New York University School of Medicine, New York, New York, United States of America; 4 Department of Medicine, Leon H. Charney Division of Cardiology, New York University School of Medicine, New York, New York, United States of America; 5 Genome Technology Center, New York University School of Medicine, New York, New York, United States of America; 6 Department of Pathology and Neurology, New York University School of Medicine, New York, New York, United States of America; Keio University, JAPAN

## Abstract

**Objective:**

Atherosclerosis is exaggerated in African American (AA) systemic lupus erythematosus (SLE) patients, with doubled cardiovascular disease (CVD) risk compared to White patients. The extent to which common Apolipoprotein L1 (APOL1) risk alleles (RA) contribute to this trend is unknown. This retrospective cohort study assessed prevalent atherosclerotic disease across APOL1 genotypes in AA SLE patients.

**Methods:**

One hundred thirteen AA SLE subjects were APOL1-genotyped and stratified as having: zero risk alleles, one risk allele, or two risk alleles. Chart review assessed CVD manifestations including abdominal aortic aneurysm, angina, carotid artery disease, coronary artery disease, myocardial infarction, peripheral vascular disease, stroke, and vascular calcifications. Associations between the genotypes and a composite endpoint defined as one or more CVD manifestations were calculated using logistic regression. Symptomatic atherosclerotic disease, excluding incidental vascular calcifications, was also assessed.

**Results:**

The 0-risk-allele, 1-risk-allele and 2-risk-allele groups, respectively, comprised 34%, 53%, and 13% of the cohort. Respectively, 13.2%, 41.7%, and 60.0% of the 0-risk allele, 1-risk-allele, and 2-risk-allele groups met the composite endpoint of atherosclerotic CVD (p = 0.001). Adjusting for risk factors–including smoking, ESRD, BMI >25 and hypertension–we observed an association between carrying one or more RA and atherosclerotic CVD (OR = 7.1; p = 0.002). For symptomatic disease, the OR was 3.5 (p = 0.02). In a time-to-event analysis, the proportion of subjects free from the composite primary endpoint, symptomatic atherosclerotic CVD, was higher in the 0-risk-allele group compared to the 1-risk-allele and 2-risk-allele groups (χ^2^ = 6.5; p = 0.04).

**Conclusions:**

Taken together, the APOL1 RAs associate with prevalent atherosclerotic CVD in this cohort of AA SLE patients, perhaps reflecting a potentiating effect of SLE on APOL1-related cardiovascular phenotypes.

## Introduction

Systemic lupus erythematosus (SLE) is an undulating inflammatory multi-organ system autoimmune disease that is associated with premature atherosclerosis and mortality [1]. SLE cardiovascular risk is further exaggerated in African American (AA) patients, with reports of 2-fold increases in cardiovascular disease (CVD) compared to White SLE patients [2]. While genetic factors contributing to increased AA risk remain elusive, recent admixture linkage studies have identified mutations in the Apolipoprotein L1 (APOL1) gene that associate with non-diabetic renal disease and CVD in homozygous carriers [3–6]. These risk alleles (RA) are common in individuals of African ancestry, in whom they also confer an evolutionary advantage for resisting African trypanosomiasis [7–9]. Consistent with the participation of APOL1 in the innate immune response, its expression is highly responsive to inflammatory signals [10]. In *ex vivo* cell culture models, gene transcription can be amplified by both Toll-like receptor (TLR) ligation and the inflammatory cytokines TNF-α, IFN-α and IFN-γ [10]. The extent to which these cytokine elevations, characteristic of SLE, influence APOL1-related atherosclerotic risk in AA SLE patients is unknown. While APOL1 associations with atherosclerotic disease have been reported in general AA populations, these associations in the context of potential penetrance-altering conditions such as chronic autoimmune disease remain to be investigated [3].

The APOL1 gene, located on chromosome 22q12.3, encodes a 4-domain protein that functions both as a minor component of circulating small high-density lipoprotein particles 3 (HDL3), and as an intracellular death signal [9]. Two mutations, G1 and G2, have been evolutionarily selected due to structural changes that promote superior resistance to *Trypanosoma brucei* [7, 9]. The third domain of human APOL1 forms an ion pore that, upon ingestion by invading trypanosomes, inserts into the organisms’ lysosomes. The resultant chloride anion influx causes lysosomal lysis and trypanosomal killing. Interestingly, the “BH3-only” domain, contained within the pore domain, appears to be divergently bioactive in human cells; when overexpressed *in vitro*, ancestral APOL1 (G0) plays a physiologic role in promoting autophagy in regressing tissues [7]. Over-expression of APOL1 may ultimately promote pore forming in mitochondrial and cell surface lipid bilayers further contributing to cytotoxicity by multiple causes including pyroptotic or inflammatory cell death [11, 12]. Variant APOL1 is cytotoxic to multiple cell types, potentially contributing to its association with increased risk of renal disease and CVD [13–15]. In AA the G1 and G2 alleles are common with minor allelic frequencies of 0.21 and 0.13 respectively [16]. These polymorphisms are virtually absent from non-ancestrally African populations including European, Japanese, and Chinese [16].

APOL1 polymorphisms have been best characterized by their associations with human chronic kidney disease (CKD); individuals carrying two RA copies in any combination (G1/G1, G1/G2, G2/G2) are considered at high risk [8, 17]. Although 95% of otherwise healthy high-risk carriers never develop renal disease, population-based studies have identified significant odds ratios (ORs) in the range of 1.3–2.5 for the association of zero or one risk allele vs two risk alleles and progressive non-diabetic renal disease [5, 18]. The impact of APOL1 genotypes appears to be highest in populations with comorbid infectious or inflammatory diseases. For example, the ORs for renal risk range from 5 in SLE collapsing glomerulonephropathy and up to 29–89 in HIV-associated nephropathy in AA and South Africans respectively [19–21]. This risk differential suggests the importance of “second hits” in the relationship between the APOL1 genotypes and human disease.

In contrast to renal disease, APOL1 polymorphisms have been inconsistently correlated with cardiovascular risk. Of five large population-based studies, analysis of three observed associations between double risk allele status and CVD with ORs of approximately 2, while two others reported no increased risk [3, 18, 22, 23]. It is possible that the inconsistencies in risk allele-ascribed cardiovascular disease in these studies are due to differences in medical comorbidities among the cohorts. However, no published study has evaluated the interaction of APOL1 genotypes and atherosclerotic disease in the context of undulating inflammation as occurs in SLE. Accordingly, we assessed differences in prevalent CVD and cardiovascular risk factors across APOL1 genotypes in a cohort of AA SLE patients.

## Patients and methods

### Study population

This study was approved by the Institutional Review Boards of New York University School of Medicine (NYUSoM) and Bellevue Hospital Center of the New York City Health and Hospitals Corporation, prior to its initiation. Individuals ≥18 years of age, of self-reported AA ancestry, and meeting at least four of the American College of Rheumatology (ACR) revised criteria for SLE were invited to participate [24]. Study subjects were not genetically related. Self-reported ancestry was confirmed by ancestry informative markers, and subjects without significant African admixture were excluded from analysis. Patients unwilling or unable to complete informed consent were excluded. Subjects were recruited between January 1, 2013 and December 31, 2016 from three high-volume SLE clinical sites. Written informed consent was obtained prior to the collection of data.

### Data collection

All subjects underwent a complete physical examination on three clinical visits, each at least six months apart, which were recorded for the purposes of this study. Subjects submitted blood samples for genetic testing, and comparisons were made across APOL1 genotypes. At each visit, SLE disease activity was measured using a hybrid version of the SELENA-Systemic Lupus Erythematosus Disease Activity Index (SELENA-SLEDAI) [25, 26]. Blood pressure measurements were taken according to the World Health Organization guidelines using automated digital blood pressure monitors outfitted by the respective clinical sites. Subjects with a history of hypertension on chart review, taking anti-hypertensive drugs for the self-reported purpose of blood pressure lowering, or who had blood pressure readings of >140 mm Hg systolic or >90 mm Hg diastolic on at least two occasions were considered hypertensive. Medical chart review included hospital and outpatient encounters, hospital discharge summaries, laboratory data, imaging and procedure reports, electrocardiographic tracings, and echocardiogram reports.

Based on chart review and patient interview, information was collected on traditional cardiovascular risk factors including age, gender, smoking history, body mass index (BMI), diabetes history (by chart review, medication history, and A1C as available), hypertension (as described above), and dyslipidemia (by chart review, medication history, LDL>130, or HDL<40). Renal-related cardiovascular risk factors collected included chronic proteinuria (>500 mg/day of urinary protein for >6 months), and estimated glomerular filtration rate (eGFR) <60 mL/min/1.73 m^2^ for >6 months (CKD-EPI formula). SLE-related cardiovascular risk factors were assessed including average prednisone dose over ≥12 months, average SLE disease activity (SLEDAI) over ≥12 months, and history of lupus nephritis. History of anti-phospholipid syndrome–defined by a history of a blood clot, one second-trimester or ≥3 first-trimester miscarriages, and positive laboratory evidence including lupus anticoagulant, IgG or IgM anti-β2 glycoprotein, or anti-cardiolipin antibody test–was also collected [27].

CVD manifestations were grouped into two master composite endpoints including ***1)*** *Non-atherosclerotic*: cardiac arrest, congestive heart failure (CHF), diagnosed arrhythmia, left ventricular hypertrophy (LVH); or ***2)*** *Atherosclerotic*: Abdominal aortic aneurysm (AAA), typical angina, carotid artery disease, myocardial infarction (MI), coronary artery disease, peripheral vascular disease (PVD), stroke, or vascular calcifications on imaging. Atherosclerotic endpoints above were then re-analyzed excluding incidental vascular calcifications to evaluate for associations with symptomatic atherosclerotic CVD (*symptomatic AsCVD*). Table 1 details the methods for assessing these manifestations.

**Table 1:** Definitions of cardiovascular manifestations.

Manifestation	Chart Review Criteria	Objective Criteria
**Cardiac arrest**	Chart history	—
**Congestive heart failure**	Chart history	• Reduced left ventricular ejection fraction ○ Echocardiogram ○ Cardiac MRI ○ Angiogram • Left ventricular diastolic dysfunction ○ Echocardiogram ○ Cardiac catheterization
**Arrhythmia**	Chart history	• Electrocardiogram evidence
**Left ventricular hypertrophy**	—	• Electrocardiogram criteria • Positive imaging ○ Echocardiogram ○ Cardiac MRI
**Typical angina**	Chart history	—
**Peripheral vascular disease**	Chart history	• Abnormal ankle-brachial index
**Abdominal aortic aneurysm**	—	• Positive Imaging ○ Abdominal ultrasonography ○ Abdominal CT ○ Abdominal MRI
**Carotid artery disease**	Clinical report of prior carotid endarterectomy	—
• **Carotid plaque**	—	• Abnormal carotid intima-media thickness by ultrasound
• **Carotid stenosis**	—	• ≥50% reduction in carotid artery lumen on imaging ○ Carotid duplex ultrasound ○ MRI angiography ○ CT angiography ○ Cerebral angiography
**Myocardial infarction**	Chart evidence of acute coronary syndrome	• Supporting electrocardiogram evidence • Elevated biomarkers on ≥3 occasions
**Vascular calcifications**	—	• Apparent on CT imaging
**Coronary artery disease**	Chart history	• Positive stress test • Prior percutaneous coronary intervention • Prior coronary artery bypass graft surgery • Positive imaging ○ Cardiac MRI ○ CT angiography
**Stroke**	Chart history	• Positive imaging ○ Brain MRI ○ Brain CT

### Apolipoprotein L1 genotyping

Genomic DNA was isolated from anti-coagulated whole blood collected in EDTA blood sample tubes using the Qiagen kit (Valencia, CA, USA) according to the manufacturer’s instructions. DNA was isolated from blood samples taken at clinical visits within 24–38 hours of blood draw. DNA isolates were stored at -80°C. Batches of 10–15 DNA samples were evaluated at a time, and quantitated using a Nanodrop-1000 spectrophotometer (Nanodrop Products, Wilmington, DE). 100 ng of genomic DNA was used as a template for conventional polymerase chain reaction (PCR). A single 300-base-pair DNA segment containing the APOL1 polymorphisms, G1 (rs73885319 and rs60910145) and G2 (rs71785313), was amplified using AmpliTaq Gold 360 DNA Polymerase (Applied Biosystems, Foster City, CA). For quality control, DNA was elongated in both forward and reverse directions. Genotypes were analyzed using the GeneWiz online platform as previously described [28].

**Fig 1:**
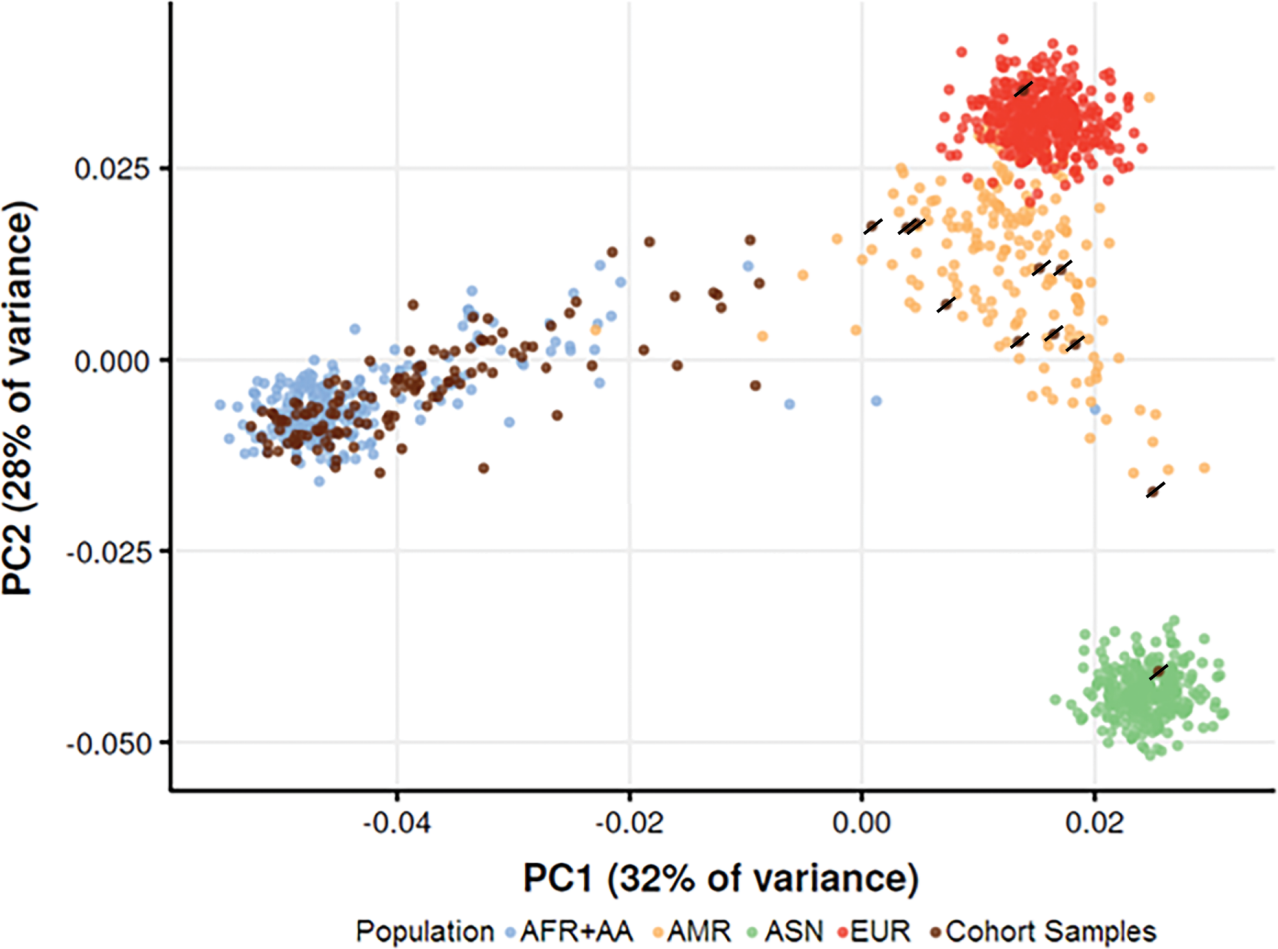
Cohort ancestral informative markers principal component analysis: Plot showing first and second principal components (PC1 and PC2 respectively). Ancestry was estimated using the 1kG Phase 1 Project containing 1,092 reference samples, including 246 samples with African (AFR) ancestry (blue), 181 with Admixed American (AMR) ancestry (orange), 286 with Asian (ASN) ancestry (green), and 379 with European (EUR) ancestry (red). Overlying African American cohort samples are shown in brown. Samples marked with a dash were excluded from analysis.

To assess the accuracy of self-reported ancestry information, a principal component analysis (PCA) was used. All 135 samples were assayed at 15,949 markers using the Infinium QC Array and integrated with the 1000 Genomes (1kG) Phase 1 project [29]. The 1kG Phase 1 project contains 1092 samples from 14 countries corresponding to four super-populations; of these, there are 246 samples with African (AFR) ancestry, 181 with Admixed American (AMR) ancestry, 286 with Asian (ASN) ancestry, and 379 with European (EUR) ancestry. To identify variants present in both the Infinium QC Array and the 1kG Phase 1 project, rs numbers associated with array probes were determined and all available 1kG Phase 1 genotypes extracted. Using PLINK (v1.90b3.43) [30], sample data from the Infinium QC Array were merged with 1kG Phase 1 genotypes and the resulting dataset subjected to quality control filters. First, any variants missing in more than 10% of samples were removed, and then any samples missing more than 10% of genotypes were removed. Finally, any variants with a minor allelic frequency less than 5% were also removed from the analysis. To ensure that the final principal component analysis was not biased due to correlation between variants, linkage disequilibrium pruning was performed [31], removing any variants within 500 kb windows with a pairwise r2 correlation greater than 0.05. These quality control measures resulted in a final dataset consisting of 2,826 variants, and no samples assayed were removed due to missing genotype information. Principal components were calculated using PLINK, and the first and second principal components were plotted against each other (Fig 1).

### Statistical analysis

An SPSS version 23 platform housed demographics, ACR criteria, interval history, medications, vital signs, physical exam, laboratory information, and disease activity assessments (SELENA-SLEDAI) [25]. Patient demographic characteristics, comorbidities, and cardiovascular risk factors were compared among genotype groups to ensure there were no statistically significant differences, using Kruskal-Wallis tests for continuous variables and Fisher’s exact test for categorical variables.

The primary outcome variables were dichotomized composite endpoints as defined above. Logistic regressions were employed to explore the association between the master composite endpoints and the APOL1 genotypes, both with regard to risk allele gene dose (zero risk alleles vs one risk allele vs two risk alleles) and in combination (0-risk-allele + 1-risk-allele groups vs two risk alleles; and zero risk alleles vs 1-risk-allele + 2-risk-allele groups). Bivariate logistic regression was performed for each potentially confounding covariate on outcome variables separately, and those with the strongest associations (p <0.10) were incorporated in the multivariate analysis. Models were also adjusted for African admixture using the Euclidean distance (√[(PC1_AA_-AveragePC1_AFR_)^2^+(PC2_AA_-AveragePC2_AFR_)^2^]) between principal components (PC) 1 and 2 of the AA samples and mean values for AFR samples. In the time-to-event analysis, data were censored at the time of death, or last study visit date as of December 31, 2016. The Log-rank test was applied to compare differences across the genotypes and a two-sided p<0.05 was considered to be statistically significant. All analyses used SAS software, version 9.3 (SAS Institute). To adjust for multiple comparators across the three genotypes, associations meeting a more stringent p value of 0.01 were also noted. A post-hoc power analysis was performed showing that sample sizes of 58 or greater would provide 95% power to detect our effect size at a 0.05% significance level.

## Results and discussion

### Characteristics of the SLE cohort

One hundred forty eight AA patients met inclusion criteria, consented to participate, and were enrolled. Each patient provided whole blood for DNA extraction, and all DNA samples met quality control measures. Twenty three patients were subsequently excluded due to missing CVD outcome data. In an additional 12 subjects, the Euclidean distances between sample PC1/PC2 and that of known African subjects were greater than 2 standard deviations above the cohort mean suggesting lack of significant African admixture. These subjects were also excluded. There were no significant differences in gender, age, CVD risk factors, average SLE activity scores, end stage renal disease (ESRD), or eGFR between the initial 148 and the final 113 subjects.

As shown in Table 2, the mean age of subjects at enrollment was 42.4 ± 13.8 years, with an average SLE disease duration of 12.8 ± 9.5 years. Ninety two percent of subjects were female, and 4.9% were of Hispanic ethnicity. A history of anti-dsDNA antibody positivity and low complement levels were present in 69% and 51% of subjects, respectively. Anti-phospholipid syndrome was present in 25% of subjects. Fifty seven percent of subjects had a history of lupus nephritis; among these the mean eGFR was 86.9 ± 45.4 mL/min/1.73 m^2^. (Raw data may be viewed in S1 Table, with abbreviations defined in S2 Table.)

**Table 2:** Patient characteristics.

	Genotype			*p* value	
	0 Risk Alleles	1 Risk Allele	2 Risk Alleles		Total
	(n = 38)	(n = 60)	(n = 15)		(n = 113)
**Demographics**					
Age (years)	43.8 ± 13.3	40.7 ± 13.9	46.2 ± 15	0.29	42.4 ± 13.8
Gender (% female)	94.7	90.0	93.3	0.69	92.0
Hispanic (%)	3.8	4.8	7.6	0.87	4.9
African Admixture*	0.017	0.013	0.012	0.17	0.016
**CVD clinical and lab values^a^**					
Systolic BP (mmHg)^d^	123 ± 13.7	123.3 ± 14	129 ± 17	0.40	124 ± 14
Diastolic BP (mmHg)^d^	74 ± 7.9	77 ± 9	79 ± 11	0.19	76 ± 9
BMI (kg/m^2^)^d^	27.9 ± 8.4	29.9 ± 8.1	26.1 ± 3.5	0.23	28.7 ± 7.9
LDL-C (mmol/L)^d^	2.7 ± 0.9	2.6 ± 0.8	2.8 ± 0.7	0.77	2.7 ± 0.8
HDL-C (mmol/L)^d^	1.4 ± 0.4	1.4 ± 0.5	1.5 ± 0.4	0.95	1.4 ± 0.4
Cholesterol (mmol/L)^d^	4.6 ± 1.0	4.9 ± 1.2	4.9 ± 1.0	0.62	4.8 ± 1.2
Triglycerides (mmol/L)^d^	1.3 ± 0.6	1.1 ± 0.6	1.3 ± 0.6	0.51	1.2 ± 0.6
A1C (x total hemoglobin)^d^	0.06 ± 0.01	0.06 ± 0.01	0.06 ± 0.01	0.76	0.06 ± 0.01
**Traditional CVD risk factors (%)**					
Smoking	23.6	21.6	20.0	0.95	22.1
Diabetes	2.8	1.7	13.3	0.09	3.5
Hypertension	50.0	68.3	80.0	0.07	73.7
Obesity	26.5	49.1	15.4	**0.02**^**b**^	37.0
Overweight	22.4	22.6	46.2	0.23	29.0
Dyslipidemia*	66.7	66.7	80.0	0.71	68.6
**SLE-related CVD risk factors^a^**					
Disease duration (years)	14.7 ± 10.1	11.3 ± 8.6	14.0 ± 11.1	0.21	12.8 ± 9.5
Nephritis (%)	55.3	60.0	47.0	0.64	56.6
ESRD (%)^c^	7.9	1.8	20.0	**0.03**^**b**^	6.2
eGFR (mL/min/1.73m^2^)^c^^d^	84.1± 47.6	97.4 ± 39.6	58.2 ± 51.3	0.12	86.9 ± 45.4
Urine protein (g/day)^c^^d^	1.5 ± 1.7	1.8 ± 1.8	2.1 ± 3.1	0.82	1.7 ± 2.0
SLEDAI^d^	3.2 ± 2.9	4.6 ± 3.7	3.1 ± 3.2	0.08	3.9 ± 3.5
C3 (mg/dL)^d^	100.3 ± 26.2	95.2 ± 29.2	101.4 ± 28.5	0.69	98.1 ± 27.8
C4 (mg/dL)^d^	23.7 ± 10.2	21.8 ± 11.1	24.6 ± 14.2	0.66	22.9 ± 11.2
Anti-dsDNA antibodies (%)	71.1	71.7	53.3	0.37	69.0 ± 46.4
APLS (%)	25.6	21.2	40.0	0.32	25.0
**Cardiovascular medications**					
Statin (% taking)	24.3	16.7	13.3	0.60	18.5
ASA (% taking)	26	26	17	0.79	24.6
ACE or ARB (% taking)	33	47	58	0.29	43.9
Anti-hypertensive (% taking)	60	65	58	0.85	62.6
**SLE medications**					
Average prednisone dose (mg)^d^	5.6 ± 9.9	8.9 ± 12.7	6.9 ± 13.7	0.39	7.6 ± 11.9
Average HCQ Dose (mg)^d^	343.8 ± 121.6	344.4 ± 139.5	302.2 ± 168.8	0.55	338.6 ± 137.5
Cyclophosphamide (% taking)	20.7	16.3	15.4	0.87	17.6
MMF (% taking)	42.0	44.2	57.1	0.63	45.4
AZA (% taking)	22.6	32.7	7.7	0.16	26.0
Belimumab (% taking)	12.9	11.5	21.4	0.63	13.4

*Abbreviations*: CVD, cardiovascular disease; BP, blood pressure; BMI, body mass index; LDL-C, low density lipoprotein cholesterol; HDL-C, high density lipoprotein cholesterol; A1C, glycated hemoglobin; ESRD, end stage renal disease; eGFR, estimated glomerular filtration rate; SLEDAI, Systemic Lupus Erythematosus Disease Activity Score; dsDNA, double-stranded DNA; APLS, anti-phospholipid syndrome; ASA, aspirin; ACE, angiotensin-converting enzyme inhibitor; ARB, angiotensin II receptor antagonist; HCQ, hydroxychloroquine; MMF, mycophenolate mofetil; AZA, azathioprine.

*African admixture as measured by the Euclidean distance between known West African subjects and African American subjects. Higher distances represent lower percent African admixture.

^a^ Values expressed as mean ± standard deviation unless otherwise indicated.

^b^ Statistically significant

^c^ Indicated values are among patients with SLE nephritis only.

^d^ Indicated values represent the mean of three measurements taken 4–6 months apart.

With regard to cardiovascular risk factors, 22% of the cohort had a greater than 10 pack-year smoking history, and hypertension was present in 74%. Only 3% of the cohort carried a diagnosis of diabetes, with mean hemoglobin A1c of 5.8 ± 1.2% (0.06 ± 0.01). Mean BMI was 28.7 ± 7.9 kg/m^2^, with 37.0% of patients obese and 29.0% overweight at the time of first visit. Dyslipidemia was present in 68.6% of patients. Average LDL-C was 106 ± 39.6 mg/dL (2.7 ± 0.8 mmol/L), HDL-C was 56.5 ± 17 mg/dL (1.4 ± 0.4 mmol/L), and total cholesterol was 186.3 ± 45.3 mg/dL (4.8 ± 1.2 mmol/L). Table 2 summarizes the cohort characteristics including traditional and SLE-related cardiovascular risk factors both as a whole and across genotypes.

### Comparisons of SLE patients across APOL1 genotypes

As shown in Table 2, 38 subjects had zero risk alleles, 60 had one risk allele, and 15 had two risk alleles. Overall the frequency of the G0 allele was 60.2%, the G1 variant was 22.1%, and the G2 variant was 17.7%; the alleles were in Hardy Weinberg Equilibrium (p = 0.69).

Comparable proportions of patients had a history of nephritis based on ACR criteria across the genotypes (zero risk alleles: 65.3%; one risk allele: 60.0%; two risk alleles: 47.0%; p = 0.64). However, among patients with nephritis, a larger percentage of the 2-risk-allele group had progressed to ESRD at the time of enrollment in this study (zero risk alleles: 7.9%, one risk allele: 1.8%; two risk alleles: 20.0%; recessive model OR = 5.7; p = 0.048). Though not statistically significant, there was a trend toward lower eGFR in 2-risk-allele nephritis subjects. The average eGFR was 84.1 ± 47.6 mL/min/1.73 m^2^ in the 0-risk-allele group; 97.4 ± 39.6 mL/min/1.73 m^2^ in the 1-risk-allele group, and 58.2 ± 51.3 mL/min/1.73 m^2^ in the 2-risk-allele group (*p* = 0.12).

SLE disease activity scores and medication histories were similar across the APOL1 genotype groups. There were no significant differences in average SLEDAI over a 12-month period (zero risk alleles: 3.2 ± 2.9; one risk allele: 4.6 ± 3.7; two risk alleles: 3.1 ± 3.2; p = 0.08). There was no difference in disease duration across genotypes (zero risk alleles: 14.7 ± 10.1 years; one risk allele: 11.3 ± 8.6 years; two risk alleles: 14.0 ± 11.1 years; p = 0.21). Supporting their role in vascular pathology, each additional risk allele increased the odds of prevalent avascular necrosis (AVN) even when controlling for prednisone dose, with 5.3% of the 0-risk-allele group, 16.7% of the 1-risk-allele group and 33.3% of the 2-risk-allele group having had a history of AVN (zero risk alleles vs one risk allele: OR = 3.9, 95% CI 0.8–19.1, *p* = 0.09; zero risk alleles vs two risk alleles: OR = 9.3, 95% CI 1.6–56.1, *p* = 0.015). There were no differences in ever or current use of cyclophosphamide, mycophenolate mofetil, hydroxychloroquine, or prednisone across the genotypes.

### Associations between the risk allele and CVD

There was an association between the percentage of subjects afflicted with *atherosclerotic CVD* and number of risk alleles (Table 3), with 13.2% of the 0-risk-allele group compared to 41.7% of the 1-risk-allele group and 60.0% of the 2-risk-allele group (*p* = 0.001*) meeting criteria. Likewise, a higher percentage of both the 2-risk-allele group (53.3%) and 1-risk-allele group (28.3%) had *symptomatic AsCVD* compared to the 0-risk-allele group (13.2%), reaching statistical significance (*p* = 0.01). By comparison, there was no significant trend between the APOL1 genotype and *non-atherosclerotic CVD* (zero risk alleles 13.2%; one risk allele 16.7%; two risk alleles 28.6%; *p* = 0.4). Table 3 details specific components of each composite endpoint across the genotype groups.

**Table 3:** Comparison of cardiovascular manifestations across APOL1 genotypes.

	0 Risk Alleles	1 Risk Allele	2 Risk Alleles	
	(n = 38)	(n = 60)	(n = 15)	*p* value
**Non-atherosclerotic CVD**	**13.2%**	**16.7%**	**28.6%**	0.4
Arrhythmia, n (%)	0 (0)	5 (8.3)	2 (13.3)	
Cardiac arrest, n (%)	1 (2.6)	2 (3.4)	0 (0)	
Congestive heart failure, n (%)	1 (2.6)	2 (3.4)	3 (20.0)	
Left ventricular hypertrophy, n (%)	4 (10.5)	5 (8.3)	3 (20.0)	
**Atherosclerotic CVD**	**13.2%**	**41.7%**	**60.0%**	**0.001**^**a**^
Abdominal aortic aneurysm, n (%)	0 (0)	0 (0.0)	0 (0)	
Angina, n (%)	2 (5.3)	16 (22.7)	3 (20.0)	
Carotid artery disease, n (%)	2 (2.6)	3 (5.0)	2 (13.3)	
Coronary artery disease, n (%)	1 (2.6)	3 (5.9)	2 (13.3)	
Myocardial infarction, n (%)	1 (2.6)	5 (8.3)	2 (13.3)	
Peripheral vascular disease, n (%)	0 (0.0)	4 (6.7)	1 (6.7)	
Stroke, n (%)	3 (7.8)	7 (11.7)	3 (20.0)	
Vascular calcifications, n (%)	0 (0)	8 (13.3)	3 (20.0)	
**Symptomatic AsCVD**	**13.2%**	**28.3%**	**53.3%**	**0.01**^**a**^

*Abbreviation*: CVD: cardiovascular disease. Symptomatic AsCVD: symptomatic atherosclerotic cardiovascular disease.

^a^ Statistically significant

**Fig 2:**
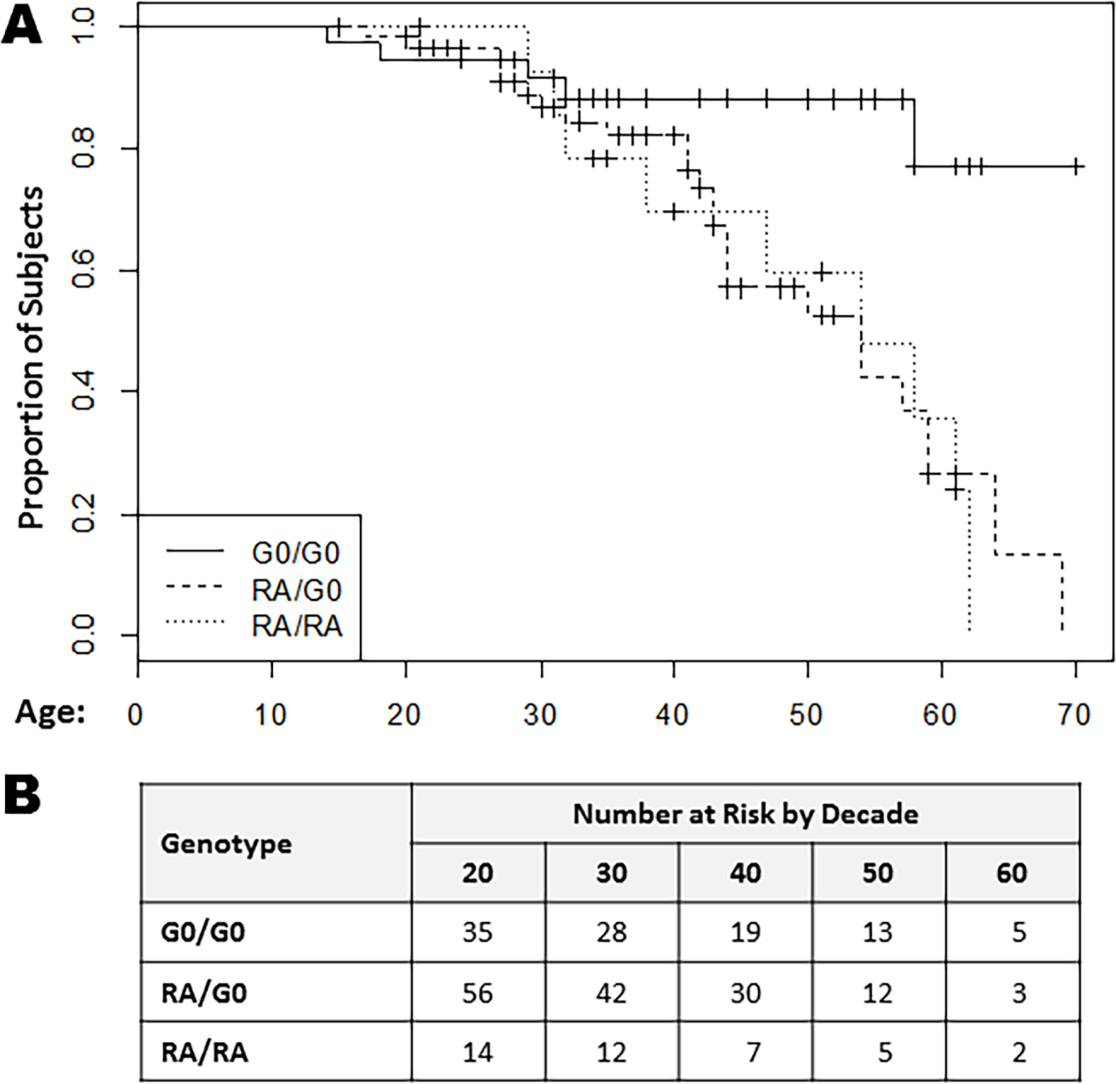
Time to atherosclerotic event analysis across APOL1 genotype. ***A)*** Time-to-event analysis for symptomatic atherosclerotic CVD as represented by Kaplan Meier Curves. The Y axis represents proportion of individuals free of the outcome, and the X axis represents subject age. Individuals were censored at latest age of follow up or death. ***B)*** The number of individuals present at each decade time point. In the 0-risk-allele group, 5/38 individuals met the outcome compared to 17/60 in the 1-risk-allele group and 8/15 in the 2-risk-allele group. Using the Log-Rank test, the proportion of subjects free from the endpoint was higher in the 0-risk-allele group compared to the 1- and 2-risk-allele groups (χ2 = 6.5; p = 0.04). Hazard Ratios (HR): for zero risk alleles vs one risk allele, HR = 4.2, 95% CI 1.6–11.0, *p* = 0.003; for zero risk alleles vs two risk alleles, HR = 4.6, 95% CI 1.5–13.8, *p* = 0.006.

In the time-to-event analysis, overall, the mean age at onset of the atherosclerotic event was 40.8 years. The proportion of individuals free from symptomatic atherosclerotic CVD over time was assessed. All other subjects were censored at age of last follow-up, with one censor due to death from heart failure. The Log-rank Test was applied to compare differences in age-to-symptomatic atherosclerotic event across APOL1 genotypes. The proportion of subjects free from the composite primary endpoint was significantly higher in the 0-risk-allele group compared to the 1-risk-allele and 2-risk-allele groups (χ^2^ = 6.5; p = 0.04). These differences were most pronounced in the third and fourth decades. These data are summarized by Kaplan-Meier curves (Fig 2).

Given the apparent intermediate rate of *atherosclerotic CVD* in the heterozygous carriers, we calculated odds ratios using two comparisons: Comparison 1, 0-risk-allele + 1-risk-allele groups vs two risk alleles; and Comparison 2, zero risk alleles vs 1-risk-allele + 2-risk-allele groups. In Comparison 1, we observed increased odds of *atherosclerotic CVD* in 2-risk-allele subjects (OR 3.4; 95% CI 1.1–10.4; *p* = 0.03). These associations remained marginally significant when adjusting for important CVD risk factors including ESRD, smoking, average prednisone dose, BMI>25, and hypertension; however, the association did not meet a more stringent significance level after adjusting for multiple comparisons (OR 3.5; 95% CI 1.0–11.9; *p* = 0.046). With regard to *symptomatic AsCVD*, both the unadjusted (OR 3.9; 95% CI 1.3–12.1; *p* = 0.02) and adjusted models (OR: 3.9; 95% CI 1.2–12.8; p = 0.02) demonstrated associations with the risk alleles though neither met the more conservative multiple comparators significance level of p = 0.016.

Using Comparison 2, we observed a significant association between carrying at least one risk allele copy and *atherosclerotic CVD* (OR 5.5; 95% CI 1.9–15.5; *p* = 0.001*) including *symptomatic AsCVD* (OR 3.2; 95% CI 1.2–8.8; *p* = 0.02). This persisted when controlling for the above-mentioned CVD risk factors with *atherosclerotic CVD* (OR 7.1, 95% CI 2.1–24.0, *p* = 0.002*) and *symptomatic AsCVD* (OR 3.5, 95% CI 1.1–11.1, *p* = 0.03). Notably, odds ratios increased with each additional risk allele. Compared to the 0-risk-allele group, having one risk allele was associated with atherosclerotic disease at an OR of 4.7 (95% CI 1.6–13.8; p = 0.005) while the OR was 9.9 for the 2-risk-allele group (95% CI 2.4–40.0; p = 0.001). A similar trend was seen in *symptomatic AsCVD* (one risk allele: OR 2.6, 95% CI 0.87–7.8, *p* = 0.09; two risk alleles: OR 7.5, 95% CI 1.9–30.1, *p* = 0.004). Likewise, upon controlling for risk factors the gene dose effect persisted in both *atherosclerotic CVD* (one risk allele: OR 6.1, 95% CI 1.8–21.4, *p* = 0.004*; two risk alleles: OR 13.4, 95% CI 2.7–67.1, *p* = 0.002*) and *symptomatic AsCVD* (one risk allele: OR 2.8, 95% CI 0.88–9.2, *p* = 0.08; two risk alleles: OR 8.4, 95% CI 1.9–37.2, *p* = 0.005*). Comparison 2 associations remain significant upon correcting for multiple comparators. Table 4 summarizes the risk models.

**Table 4:** Adjusted odds ratios of atherosclerotic cardiovascular disease across genotype groups.

	0 Risk Alleles+ 1 Risk Allele	0 Risk Alleles	0 Risk Alleles	0 Risk Alleles
	vs	vs	vs	vs
	2 Risk Alleles	1 Risk Allele	2 Risk Alleles	1 Risk Allele+ 2 Risk Alleles
**AsCVD**	OR: 3.5	OR: 6.1	OR: 13.4	OR: 7.1
	95% CI: 1.0–11.9	95% CI: 1.8–21.4	95% CI: 2.7–67.1	95% CI: 2.1–24.0
	*p* = **0.046**^**a**^	*p* = **0.004**^**b**^	***p* = 0.002**^**b**^	***p* = 0.002**^**b**^
**Symptomatic AsCVD**	OR: 3.9	OR: 2.8	OR: 8.4	OR: 3.5
	95% CI: 1.2–12.8	95% CI: 0.88–9.2	95% CI: 1.9–37.2	95% CI: 1.1–11.1
	***p* = 0.02**^**a**^	*p* = 0.08	***p* = 0.005**^**b**^	***p* = 0.03**^**a**^

*Abbreviations*: AsCVD, atherosclerotic cardiovascular disease; CI, confidence interval; OR, odds ratio.

^a^ Statistically significant

^b^ Statistically significant controlling for multiple comparators

To determine the differential effects of the G1 vs G2 allele on *atherosclerotic* CVD, logistic regressions for Comparison 2 were repeated both in subjects with G1 alleles only (zero risk alleles n = 38 vs one, G0/G1, or two risk alleles, G1/G1 n = 38) and in subjects with G2 alleles only (zero risk alleles n = 38 vs one, G0/G2, or two, G2/G2 n = 29). Compound heterozygous subjects (G1/G2) were excluded from this analysis. This model was adjusted for the above-mentioned covariates. Associations between *atherosclerotic* CVD and the RA were stronger in G2 carriers as compared to G1 carriers with odds ratios of 7.3 (95% CI 1.9–27.9, *p* = 0.004*) and 3.9 (95% CI 1.1–14.4, *p* = 0.04), respectively. Comparison 2 in the context of atherosclerotic CVD was used due to our being underpowered to determine differences in Comparison 1.

There were no associations between the risk allele and non-atherosclerotic manifestations. Specifically, in Comparison 1, there were no increased odds in either the uncontrolled (OR 1.9, 95% CI 0.5–7.1, p = 0.3) or controlled models (OR 2.2, 95% CI 0.4–13.2, p = 0.4). Likewise, in Comparison 2, we observed no trends towards increased non-atherosclerotic disease whether or not covariates were controlled (uncontrolled: OR 1.9, 95% CI 0.6–5.7, p = 0.25; controlled: OR 2.4, 95% CI 0.5–11.8, p = 0.28).

We aimed to determine the extent to which APOL1 polymorphisms associate with prevalent cardiovascular disease in an SLE cohort. To the best of our knowledge, this is the first study to evaluate cardiovascular APOL1 associations in autoimmune patients. There was a relationship between the risk allele and the percentage of patients affected by atherosclerotic disease, supporting our hypothesis that the risk allele confers a propensity toward vascular dysfunction. As a comparator, there were no associations between risk alleles and non-atherosclerotic CVD manifestations. Those with one risk allele demonstrated an intermediate rate of CVD, suggesting a possible importance of gene dose. Not only was CVD more prevalent in variant carriers, but atherosclerotic events tended to occur at earlier ages in both 1-risk-allele and 2-risk-allele carriers. Interestingly, the associations were strongest in G2 versus G1 allele carriers potentially suggesting a differential effect of the alleles on cardiovascular vs renal disease. The kidney literature supports stronger associations between the G1 allele and HIV-associated nephropathy in both African Americans and South Africans [20, 21] whereas G2 alleles have been reported to be more strongly associated with cardiovascular disease [3].

As reported in the literature, APOL1 polymorphisms associate with progressive non-diabetic, proteinuric renal disease and ESRD [18]. Our study replicated these results, demonstrating an OR = 5.7 for ESRD among SLE nephritis patients with two risk alleles compared with one or no risk alleles. Of note, there was no relationship observed between the risk alleles and prevalent SLE nephritis at the time of enrollment, although homozygous risk allele carrier status was associated with progressive renal insufficiency. Though the differences did not achieve statistical significance, the 2-risk-allele group showed a trend towards lower eGFR, compared to patients with zero or one risk alleles. These findings reflect the accepted recessive inheritance pattern of renal risk [5].

This study is not without limitations. Though adequately powered, the small sample size particularly with regard to the 2-risk-allele group was a major constraint. Larger replication studies will be needed to determine the differential effects of each polymorphism individually (G1 or G2) on cardiovascular phenotypes, eliminate the need for composite endpoints, and determine extent of APOL1 genotype influence on other indicators of SLE damage. Though all subjects were recruited from one institution, the diverse clinical sites ensured that patients from a wide variety of socioeconomic backgrounds were included. However, it is acknowledged that regional bias might influence the generalizability of these results. Finally, the retrospective design may have introduced information bias, as the results would have been contingent upon the clinical record quality; however, this bias would be expected to affect all genotype groups equally.

Given the recessive phenotype consistently reported in renal disease (e.g., effect only in patients with two risk alleles), our results demonstrating intermediate atherosclerotic risk in heterozygote carriers were unexpected. Because APOL1 polymorphisms are not loss-of-function mutations, but more likely gain-of-function mutations, the classic recessive inheritance model may not hold for all risk traits [17]. Further, the variable gene penetrance in homozygous carriers appears to be contingent upon “second hits” such as HIV infection, focal segmental glomerulosclerosis (FSGS), or SLE (proinflammatory state), where the consequence of carrying the risk allele is exaggerated [19, 32, 33]. It is likely that disease-specific conditions interact with the risk alleles to confer an injurious effect. In primary cell culture models, APOL1 variants require overexpression to reach the threshold for toxicity [34, 35]. APOL1 gene transcription can be amplified *ex vivo* by both TLR ligation and inflammatory cytokines including TNF-α and IFN-α [10]. These pathways are integral to SLE pathogenesis and likely lead to excess APOL1 expression and increased variant protein burden in risk allele carriers.

An alternative hypothesis is that APOL1 risk alleles increase CVD risk by promoting IL-6 signaling pathways known to be atherogenic in SLE patients. Recent *in vitro* studies indicate that high variant APOL1 expression promotes internalization and degradation of membrane-associated gp130 in human embryonic kidney cell lines [34]. gp130 is widely expressed and is a major mechanism by which activated IL-6 complexes are degraded *in vivo* [36]. Thus, variant APOL1 could indirectly promote increased serum IL-6 among risk allele carriers by degrading gp130. Consistent with this possibility, Sampson et al have reported increased IL-6-type cytokine signaling in renal biopsies from 2-risk-allele but not 0-risk-allele carriers [37]. Elevated serum IL-6 is a known cardiovascular risk factor in SLE patients and has been associated with both coronary artery calcification and atherosclerotic disease [1]. In SLE, increased IL-6 also correlates with dyslipidemia characterized by high triglycerides and low HDL [38].

Given the higher observed prevalence of CVD in African Americans, multiple large cohort studies have been interrogated for APOL1 risk allele associations yielding mixed results. In evaluating both the Jackson Heart Study (JHS; n = 1,959) and The Women’s Health Initiative (WHI), Ito et al showed an OR = 2 for cardiovascular events among 2-risk-allele carriers (*p* <0.001) [3]. Likewise, Mukamal et al showed lower ankle-brachial indexes and an 80% higher risk of myocardial infarction in 2-risk-allele carriers, who on average had a 3-year reduction in median overall survival compared to APOL1 low-risk African Americans and European Americans [23]. In contrast, noting that the African American Study of Kidney Disease and Hypertension (AASK) trial showed no such CVD association, Langefeld et al evaluated 2,571 AAs enrolled in the Systolic Blood Pressure Intervention Trial (SPRINT) and found no CVD association [22]. These disparate findings may be ascribed, in part, to differences in baseline comorbidities across the cohorts. Both the JHS and WHI included a heterogeneous group of patients with diabetes, stroke, chronic kidney disease, autoimmune disease, and chronic vascular disease [3]. In contrast, both the AASK and the SPRINT trials had more stringent criteria excluding individuals receiving immunosuppressive therapy or with chronic autoimmune or infectious diseases such as SLE or HIV [22]. Thus, both of these latter trials eliminated patients with concomitant comorbidities that might potentiate the risk allele phenotypes [22].

CVD is widely recognized as the leading cause of late morbidity and mortality in SLE patients, who have up to a 50-fold increased risk compared to age-matched controls [1]. With accelerated damage accrual and reduced survival, AA SLE patients have the greatest burden of CVD [2, 39]. The chronic inflammation of SLE plays an important role in each step of atherogenesis from endothelial dysfunction to plaque formation and rupture [40]. Inflammation may be of particular importance in AAAPOL1 risk allele carriers as suggested by the current study. These inquiries may aid in identifying ethnically-determined genetic risk factors for CVD in AA patients, and in turn elucidate the mechanisms by which APOL1 risk genotypes contribute to human disease.

## Supporting information

S1 Table Cardiovascular disease (CVD) data set. (PDF)

S2 Table Definitions of data bank abbreviations used in S1 Table. (PDF)
