# Supplementary material for: Apolipoprotein L1 risk variants associate with prevalent atherosclerotic disease in African American systemic lupus erythematosus patients
Source: PLoS One. 2017 Aug 29;12(8):e0182483. doi: 10.1371/journal.pone.0182483 (PMC5574561; doi:10.1371/journal.pone.0182483)
Supplement: S1 Table — (PDF) [file pone.0182483.s001.pdf]

S1 Table. Cardiovascular Disease (CVD) data set.

D1_PT_ID	D1_Age	D1_Sex	D2_DM	D2_smoking	D2_HTN	D2_BMI	D2_Statin	D3_AVN	D3_Nephritis	D3_APS	D3_ESRD	D4_cardiac_arrest	D4_arrythmia	D4_LVH	D4_CHF	D5_AAA	D5_Stroke	D5_CEA	D5_carotid_stenosis	D5_Posit_Stress	D5_angina	D5_MI	D5_CA_Revasc	D5_CA_Calc	D5_Vasc_Calc	D5_PVD	D6_TE	D8_number_risk_alleles	D8_APOL1_status
5004	58	1.00	0.00	1.00	1.00	23.50	0.00	1.00	1.00	0.00	0.00	0.00	0.00	0.00	0.00	0.00	0.00	0.00	0.00	0.00	1.00	1.00	0.00	0.00	0.00	0.00	1.00	2.00	5.00
5007	33	1.00	0.00	0.00	0.00	60.63	0.00	0.00	1.00	1.00	0.00	0.00	0.00	0.00	0.00	0.00	0.00	0.00	0.00	0.00	0.00	0.00	0.00	0.00	0.00	0.00	1.00	0.00	0.00
5009	40	1.00	0.00	0.00	1.00	25.88	0.00	0.00	1.00	0.00	0.00	n/a	0.00	0.00	0.00	0.00	0.00	0.00	0.00	0.00	0.00	0.00	0.00	0.00	0.00	0.00	0.00	1.00	2.00
5015	37	1.00	0.00	1.00	1.00	25.78	1.00	0.00	1.00	0.00	0.00	0.00	0.00	0.00	0.00	0.00	1.00	0.00	0.00	0.00	0.00	0.00	0.00	0.00	0.00	0.00	0.00	1.00	2.00
5024	50	1.00	0.00	0.00	0.00	19.77	0.00	0.00	1.00	0.00	0.00	0.00	0.00	0.00	0.00	0.00	0.00	0.00	0.00	0.00	0.00	0.00	0.00	0.00	0.00	0.00	1.00	0.00	0.00
5043	34	1.00	0.00	0.00	1.00	28.88	0.00	0.00	0.00	0.00	0.00	0.00	0.00	0.00	0.00	0.00	0.00	0.00	0.00	0.00	0.00	0.00	0.00	0.00	0.00	0.00	0.00	2.00	5.00
5044	29	1.00	0.00	0.00	1.00	23.50	1.00	1.00	1.00	0.00	0.00	0.00	0.00	0.00	0.00	0.00	0.00	0.00	0.00	0.00	1.00	0.00	0.00	0.00	0.00	0.00	0.00	0.00	0.00
5046	61	1.00	0.00	0.00	1.00	20.45	0.00	0.00	1.00	0.00	1.00	0.00	0.00	1.00	0.00	0.00	0.00	0.00	0.00	0.00	0.00	0.00	0.00	0.00	0.00	0.00	0.00	0.00	0.00
5052	44	0.00	0.00	1.00	1.00	29.50	1.00	0.00	0.00	0.00	0.00	1.00	1.00	1.00	0.00	0.00	1.00	0.00	0.00	1.00	1.00	1.00	0.00	0.00	0.00	1.00	0.00	1.00	1.00
5056	51	0.00	0.00	0.00	1.00	22.60	0.00	0.00	0.00	0.00	0.00	0.00	0.00	0.00	0.00	0.00	0.00	0.00	0.00	0.00	0.00	0.00	0.00	0.00	0.00	0.00	0.00	1.00	1.00
5062	38	1.00	0.00	0.00	1.00	21.50	0.00	1.00	1.00	0.00	0.00	0.00	0.00	0.00	0.00	0.00	0.00	0.00	0.00	0.00	0.00	0.00	0.00	0.00	0.00	0.00	0.00	1.00	2.00
5069	40	1.00	0.00	0.00	1.00	27.70	0.00	0.00	0.00	0.00	0.00	0.00	0.00	0.00	0.00	0.00	0.00	0.00	0.00	0.00	0.00	0.00	0.00	0.00	0.00	0.00	0.00	2.00	3.00
5071	62	0.00	0.00	1.00	1.00	22.16	0.00	0.00	1.00	1.00	0.00	0.00	0.00	1.00	0.00	0.00	0.00	0.00	0.00	0.00	0.00	0.00	0.00	0.00	1.00	0.00	0.00	2.00	3.00
5072	35	1.00	0.00	1.00	1.00	25.00	1.00	0.00	1.00	0.00	0.00	0.00	0.00	0.00	0.00	0.00	0.00	0.00	0.00	0.00	0.00	0.00	0.00	0.00	0.00	0.00	1.00	0.00	0.00
5079	27	1.00	0.00	0.00	1.00	31.08	0.00	0.00	1.00	0.00	0.00	0.00	0.00	0.00	0.00	0.00	0.00	0.00	0.00	0.00	0.00	0.00	0.00	0.00	0.00	0.00	0.00	1.00	1.00
5087	28	1.00	0.00	0.00	0.00	19.38	0.00	0.00	0.00	1.00	0.00	0.00	0.00	0.00	0.00	0.00	0.00	0.00	0.00	0.00	0.00	0.00	0.00	0.00	0.00	0.00	0.00	0.00	0.00
5091	62	1.00	1.00	0.00	1.00	29.20	1.00	0.00	0.00	0.00	0.00	0.00	0.00	0.00	0.00	0.00	0.00	0.00	0.00	0.00	0.00	0.00	0.00	0.00	0.00	0.00	0.00	0.00	0.00
5094	43	1.00	0.00	1.00	1.00	41.90	1.00	0.00	1.00	0.00	0.00	0.00	0.00	0.00	0.00	0.00	0.00	0.00	0.00	0.00	1.00	0.00	0.00	1.00	1.00	0.00	1.00	1.00	2.00
5096	45	1.00	0.00	0.00	1.00	n/a	0.00	1.00	1.00	0.00	0.00	0.00	0.00	0.00	0.00	0.00	0.00	0.00	0.00	0.00	0.00	0.00	0.00	0.00	0.00	0.00	1.00	1.00	1.00
5111	44	1.00	0.00	0.00	0.00	25.11	0.00	1.00	0.00	1.00	0.00	0.00	0.00	0.00	0.00	0.00	0.00	0.00	0.00	0.00	0.00	0.00	0.00	0.00	0.00	0.00	0.00	0.00	0.00
5112	50	1.00	0.00	1.00	1.00	31.08	0.00	0.00	1.00	0.00	0.00	0.00	0.00	0.00	0.00	0.00	0.00	0.00	0.00	0.00	1.00	0.00	0.00	0.00	0.00	0.00	0.00	1.00	1.00
5113	54	1.00	0.00	1.00	1.00	n/a	0.00	0.00	0.00	0.00	0.00	0.00	0.00	0.00	0.00	0.00	0.00	0.00	0.00	0.00	0.00	0.00	0.00	0.00	0.00	0.00	0.00	0.00	0.00
5119	31	1.00	0.00	0.00	0.00	n/a	1.00	1.00	0.00	1.00	0.00	0.00	0.00	0.00	0.00	0.00	0.00	0.00	0.00	1.00	1.00	1.00	1.00	1.00	1.00	1.00	1.00	1.00	2.00
5140	49	1.00	0.00	0.00	1.00	22.57	0.00	0.00	0.00	0.00	0.00	0.00	0.00	1.00	0.00	0.00	1.00	0.00	0.00	0.00	0.00	0.00	0.00	0.00	0.00	0.00	0.00	1.00	1.00
5144	50	1.00	0.00	0.00	0.00	21.70	0.00	0.00	0.00	0.00	0.00	0.00	0.00	0.00	0.00	0.00	0.00	0.00	0.00	0.00	0.00	0.00	0.00	0.00	0.00	1.00	1.00	2.00	4.00
5145	59	0.00	0.00	1.00	1.00	22.26	1.00	0.00	1.00	0.00	0.00	0.00	0.00	1.00	1.00	0.00	1.00	0.00	0.00	1.00	1.00	1.00	1.00	0.00	0.00	0.00	0.00	0.00	0.00
5151	42	1.00	0.00	0.00	0.00	30.86	0.00	0.00	1.00	1.00	0.00	0.00	0.00	0.00	0.00	0.00	0.00	0.00	0.00	0.00	0.00	0.00	0.00	0.00	0.00	0.00	0.00	1.00	1.00
5152	45	0.00	0.00	1.00	1.00	30.00	0.00	0.00	1.00	0.00	0.00	0.00	0.00	1.00	0.00	0.00	0.00	0.00	0.00	0.00	1.00	1.00	0.00	0.00	0.00	1.00	1.00	1.00	2.00
5154	42	1.00	0.00	0.00	1.00	26.30	1.00	0.00	1.00	0.00	0.00	0.00	0.00	0.00	0.00	0.00	0.00	0.00	1.00	0.00	0.00	0.00	0.00	0.00	0.00	0.00	0.00	0.00	0.00
5165	45	1.00	0.00	1.00	1.00	48.76	0.00	0.00	0.00	1.00	0.00	0.00	1.00	0.00	0.00	0.00	0.00	0.00	0.00	0.00	1.00	0.00	0.00	0.00	0.00	0.00	0.00	1.00	1.00
5172	44	1.00	0.00	0.00	1.00	n/a	1.00	0.00	1.00	0.00	0.00	0.00	0.00	0.00	0.00	0.00	1.00	0.00	0.00	0.00	0.00	0.00	0.00	0.00	0.00	0.00	0.00	0.00	0.00
5181	62	1.00	0.00	0.00	1.00	34.50	1.00	0.00	0.00	0.00	0.00	0.00	0.00	0.00	0.00	0.00	0.00	0.00	1.00	0.00	1.00	0.00	0.00	0.00	1.00	0.00	1.00	1.00	1.00
5194	56	1.00	0.00	0.00	1.00	n/a	0.00	0.00	0.00	0.00	0.00	0.00	0.00	0.00	0.00	0.00	0.00	0.00	1.00	0.00	1.00	0.00	0.00	0.00	0.00	0.00	0.00	2.00	5.00
5201	70	1.00	0.00	0.00	1.00	n/a	0.00	0.00	0.00	0.00	0.00	0.00	0.00	0.00	0.00	0.00	0.00	0.00	0.00	0.00	0.00	0.00	0.00	0.00	0.00	0.00	0.00	0.00	0.00
5204	61	1.00	0.00	0.00	1.00	n/a	1.00	0.00	1.00	0.00	0.00	0.00	0.00	0.00	0.00	0.00	0.00	0.00	0.00	0.00	0.00	0.00	0.00	0.00	0.00	0.00	1.00	0.00	0.00
5205	72	1.00	0.00	0.00	1.00	n/a	0.00	0.00	0.00	0.00	0.00	0.00	0.00	0.00	0.00	0.00	1.00	0.00	0.00	0.00	1.00	0.00	0.00	0.00	1.00	0.00	0.00	1.00	2.00
5211	38	1.00	0.00	1.00	1.00	30.40	1.00	1.00	1.00	0.00	0.00	0.00	0.00	1.00	1.00	0.00	0.00	0.00	0.00	0.00	1.00	0.00	0.00	0.00	0.00	0.00	1.00	2.00	3.00
5213	52	1.00	0.00	0.00	0.00	43.40	0.00	0.00	0.00	0.00	0.00	0.00	0.00	0.00	0.00	0.00	0.00	0.00	0.00	0.00	0.00	0.00	0.00	0.00	0.00	0.00	0.00	1.00	1.00
5240	59	0.00	0.00	0.00	1.00	33.40	0.00	1.00	1.00	0.00	0.00	0.00	0.00	0.00	0.00	0.00	1.00	0.00	0.00	0.00	0.00	0.00	0.00	0.00	0.00	0.00	0.00	1.00	2.00
5248	57	1.00	0.00	1.00	0.00	24.61	0.00	0.00	1.00	0.00	0.00	0.00	0.00	1.00	0.00	0.00	0.00	0.00	0.00	0.00	0.00	0.00	0.00	0.00	0.00	0.00	1.00	0.00	0.00
5261	28	1.00	0.00	1.00	1.00	27.60	0.00	0.00	1.00	0.00	0.00	0.00	0.00	0.00	0.00	0.00	0.00	0.00	0.00	0.00	0.00	0.00	0.00	0.00	0.00	1.00	0.00	1.00	2.00
5281	63	1.00	0.00	0.00	1.00	27.50	1.00	0.00	0.00	1.00	0.00	0.00	0.00	0.00	0.00	0.00	1.00	0.00	0.00	0.00	0.00	0.00	0.00	0.00	0.00	0.00	0.00	2.00	5.00
5331	69	1.00	0.00	0.00	1.00	30.88	0.00	0.00	1.00	0.00	0.00	0.00	0.00	0.00	0.00	0.00	0.00	0.00	0.00	0.00	1.00	0.00	0.00	0.00	0.00	0.00	0.00	1.00	2.00
5336	61	1.00	1.00	0.00	1.00	27.60	0.00	1.00	0.00	0.00	0.00	0.00	0.00	0.00	0.00	0.00	0.00	0.00	0.00	0.00	0.00	0.00	0.00	0.00	0.00	0.00	0.00	2.00	5.00
5363	22	1.00	0.00	0.00	1.00	25.29	0.00	1.00	1.00	1.00	0.00	0.00	0.00	0.00	0.00	0.00	0.00	0.00	0.00	0.00	0.00	0.00	0.00	0.00	0.00	0.00	1.00	1.00	2.00
5371	45	1.00	0.00	0.00	0.00	33.00	1.00	0.00	1.00	0.00	0.00	0.00	0.00	0.00	0.00	0.00	1.00	0.00	0.00	0.00	0.00	0.00	0.00	0.00	1.00	0.00	1.00	1.00	1.00
5403	40	1.00	0.00	0.00	1.00	35.61	0.00	0.00	1.00	0.00	0.00	0.00	0.00	0.00	0.00	0.00	0.00	0.00	0.00	0.00	0.00	0.00	0.00	0.00	0.00	0.00	0.00	1.00	1.00
5408	54	1.00	0.00	0.00	0.00	37.87	0.00	0.00	0.00	0.00	0.00	0.00	0.00	0.00	0.00	0.00	0.00	0.00	0.00	0.00	0.00	0.00	0.00	0.00	0.00	0.00	0.00	0.00	0.00
5410	33	1.00	0.00	0.00	1.00	18.20	0.00	0.00	1.00	0.00	0.00	0.00	0.00	0.00	0.00	0.00	0.00	0.00	0.00	0.00	0.00	0.00	0.00	0.00	0.00	0.00	0.00	1.00	2.00
5417	32	1.00	0.00	0.00	1.00	22.50	0.00	0.00	1.00	0.00	1.00	0.00	0.00	1.00	1.00	0.00	1.00	0.00	0.00	0.00	0.00	0.00	0.00	1.00	0.00	0.00	0.00	2.00	5.00
5427	38	1.00	0.00	0.00	0.00	35.26	0.00	0.00	0.00	0.00	0.00	0.00	0.00	0.00	0.00	0.00	0.00	0.00	0.00	0.00	0.00	0.00	0.00	0.00	0.00	0.00	0.00	0.00	0.00
5439	38	1.00	0.00	0.00	1.00	38.86	0.00	0.00	1.00	1.00	1.00	0.00	0.00	0.00	0.00	0.00	0.00	0.00	0.00	0.00	0.00	0.00	0.00	0.00	0.00	0.00	0.00	1.00	1.00
5444	63	1.00	0.00	1.00	1.00	24.48	0.00	0.00	1.00	0.00	0.00	0.00	0.00	0.00	0.00	0.00	0.00	0.00	0.00	0.00	0.00	0.00	0.00	0.00	0.00	0.00	0.00	0.00	0.00
5448	24	1.00	0.00	0.00	0.00	19.92	0.00	0.00	0.00	0.00	0.00	0.00	0.00	0.00	0.00	0.00	0.00	0.00	0.00	0.00	0.00	0.00	0.00	0.00	0.00	0.00	0.00	1.00	1.00
5450	64	1.00	0.00	1.00	1.00	31.23	0.00	0.00	0.00	0.00	0.00	0.00	0.00	1.00	0.00	0.00	0.00	0.00	0.00	0.00	1.00	0.00	0.00	0.00	0.00	0.00	0.00	1.00	1.00
5451	32	1.00	0.00	0.00	1.00	n/a	0.00	0.00	1.00	1.00	0.00	0.00	1.00	0.00	1.00	0.00	0.00	0.00	0.00	0.00	1.00	0.00	0.00	0.00	0.00	0.00	1.00	1.00	2.00
5454	41	1.00	0.00	0.00	0.00	n/a	0.00	0.00	0.00	0.00	0.00	0.00	0.00	0.00	0.00	0.00	0.00	0.00	0.00	0.00	0.00	0.00	0.00	0.00	0.00	0.00	0.00	1.00	1.00
5459	43	1.00	0.00	0.00	1.00	29.48	0.00	1.00	1.00	1.00	0.00	0.00	0.00	0.00	0.00	0.00	0.00	0.00	0.00	0.00	0.00	0.00	0.00	0.00	0.00	0.00	0.00	1.00	1.00
5471	48	1.00	0.00	0.00	1.00	40.44	0.00	1.00	1.00	0.00	0.00	0.00	0.00	0.00	0.00	0.00	0.00	0.00	0.00	0.00	0.00	0.00	0.00	0.00	0.00	0.00	0.00	1.00	2.00
5476	35	1.00	0.00	0.00	1.00	19.60	0.00	0.00	1.00	1.00	0.00	0.00	1.00	0.00	0.00	0.00	1.00	0.00	0.00	0.00	0.00	0.00	0.00	0.00	0.00	0.00	0.00	1.00	2.00
5484	18	1.00	0.00	0.00	0.00	17.74	0.00	0.00	1.00	1.00	0.00	0.00	0.00	0.00	0.00	0.00	1.00	0.00	0.00	0.00	0.00	0.00	0.00	0.00	0.00	0.00	0.00	0.00	0.00
5509	44	1.00	0.00	1.00	1.00	40.44	0.00	0.00	0.00	0.00	0.00	0.00	0.00	0.00	0.00	0.00	0.00	0.00	0.00	0.00	1.00	0.00	0.00	0.00	0.00	0.00	0.00	1.00	1.00
5515	21	1.00	0.00	0.00	0.00	20.51	0.00	0.00	1.00	0.00	0.00	0.00	0.00	0.00	0.00	0.00	0.00	0.00	0.00	0.00	0.00	0.00	0.00	0.00	0.00	0.00	0.00	2.00	3.00
5516	34	1.00	0.00	0.00	0.00	33.70	n/a	0.00	0.00	0.00	0.00	0.00	0.00	0.00	0.00	0.00	0.00	0.00	0.00	0.00	0.00	0.00	0.00	0.00	0.00	0.00	0.00	0.00	0.00

D1_PT_ID	D1_Age	D1_Sex	D2_DM	D2_smoking	D2_HTN	D2_BMI	D2_Statin	D3_AVN	D3_Nephritis	D3_APS	D3_ESRD	D4_cardiac_arrest	D4_arrythmia	D4_LVH	D4_CHF	D5_AAA	D5_Stroke	D5_CEA	D5_carotid_stenosis	D5_Posit_Stress	D5_angina	D5_MI	D5_CA_Revasc	D5_CA_Calc	D5_Vasc_Calc	D5_PVD	D6_TE	D8_number_risk_alleles	D8_APOL1_status
5529	30	1.00	0.00	0.00	0.00	20.83	0.00	0.00	0.00	0.00	0.00	0.00	0.00	0.00	0.00	0.00	0.00	0.00	0.00	0.00	0.00	0.00	0.00	0.00	0.00	0.00	0.00	1.00	1.00
5530	47	1.00	0.00	0.00	1.00	19.09	0.00	0.00	1.00	0.00	0.00	0.00	0.00	0.00	0.00	0.00	0.00	0.00	0.00	0.00	0.00	0.00	0.00	0.00	0.00	0.00	0.00	0.00	0.00
5532	61	1.00	0.00	0.00	0.00	29.90	0.00	0.00	0.00	0.00	0.00	0.00	0.00	0.00	0.00	0.00	0.00	0.00	0.00	0.00	0.00	0.00	0.00	0.00	0.00	0.00	0.00	1.00	2.00
5535	28	1.00	0.00	0.00	1.00	26.90	0.00	0.00	1.00	0.00	0.00	0.00	0.00	0.00	0.00	0.00	0.00	0.00	0.00	0.00	0.00	0.00	0.00	0.00	0.00	0.00	0.00	1.00	2.00
5536	36	1.00	0.00	1.00	1.00	22.30	0.00	0.00	1.00	1.00	0.00	0.00	0.00	0.00	0.00	0.00	0.00	0.00	0.00	0.00	0.00	0.00	0.00	0.00	0.00	0.00	1.00	1.00	1.00
5539	51	1.00	1.00	0.00	1.00	n/a	0.00	1.00	1.00	0.00	1.00	0.00	0.00	0.00	0.00	0.00	0.00	0.00	0.00	0.00	0.00	0.00	0.00	0.00	0.00	0.00	0.00	2.00	4.00
5543	21	0.00	0.00	0.00	1.00	21.90	0.00	0.00	1.00	0.00	0.00	0.00	1.00	0.00	0.00	0.00	0.00	0.00	0.00	0.00	0.00	0.00	0.00	0.00	0.00	0.00	0.00	1.00	1.00
5546	42	1.00	0.00	0.00	1.00	33.50	0.00	0.00	0.00	0.00	0.00	0.00	0.00	0.00	0.00	0.00	0.00	0.00	0.00	0.00	0.00	0.00	0.00	0.00	0.00	0.00	0.00	0.00	0.00
5547	44	0.00	0.00	1.00	0.00	21.10	0.00	0.00	0.00	0.00	0.00	0.00	0.00	0.00	0.00	0.00	0.00	0.00	0.00	0.00	0.00	0.00	0.00	0.00	0.00	0.00	0.00	0.00	0.00
5552	44	1.00	0.00	0.00	1.00	35.20	1.00	0.00	1.00	0.00	0.00	0.00	0.00	0.00	0.00	0.00	0.00	0.00	0.00	0.00	0.00	0.00	0.00	0.00	0.00	0.00	0.00	1.00	2.00
5554	28	1.00	0.00	0.00	0.00	27.18	0.00	0.00	1.00	0.00	0.00	0.00	0.00	0.00	0.00	0.00	0.00	0.00	0.00	0.00	0.00	0.00	0.00	0.00	0.00	0.00	0.00	1.00	2.00
5556	27	1.00	0.00	0.00	1.00	26.10	0.00	0.00	1.00	0.00	0.00	0.00	0.00	0.00	0.00	0.00	0.00	0.00	0.00	0.00	1.00	0.00	0.00	0.00	0.00	0.00	0.00	1.00	1.00
5557	37	1.00	0.00	1.00	0.00	n/a	0.00	0.00	0.00	1.00	0.00	0.00	0.00	0.00	0.00	0.00	0.00	0.00	0.00	0.00	0.00	0.00	0.00	0.00	0.00	0.00	0.00	1.00	1.00
5558	49	1.00	0.00	0.00	1.00	30.40	0.00	0.00	0.00	0.00	0.00	0.00	0.00	0.00	0.00	0.00	0.00	0.00	0.00	0.00	0.00	0.00	0.00	0.00	0.00	0.00	0.00	1.00	1.00
5566	38	1.00	0.00	1.00	0.00	23.40	0.00	0.00	1.00	0.00	1.00	1.00	0.00	0.00	0.00	0.00	0.00	0.00	0.00	0.00	0.00	0.00	0.00	0.00	0.00	0.00	0.00	0.00	0.00
5567	47	1.00	0.00	1.00	0.00	29.23	0.00	0.00	0.00	1.00	0.00	0.00	0.00	0.00	0.00	0.00	0.00	0.00	0.00	0.00	0.00	0.00	0.00	0.00	0.00	0.00	1.00	0.00	0.00
5576	15	1.00	0.00	0.00	1.00	n/a	0.00	0.00	1.00	0.00	0.00	0.00	0.00	0.00	0.00	0.00	0.00	0.00	0.00	0.00	0.00	0.00	0.00	0.00	0.00	0.00	0.00	1.00	2.00
5580	52	1.00	0.00	0.00	1.00	28.30	0.00	0.00	1.00	0.00	0.00	0.00	0.00	1.00	0.00	0.00	0.00	0.00	0.00	0.00	0.00	0.00	0.00	0.00	0.00	0.00	1.00	0.00	0.00
5581	30	1.00	0.00	0.00	1.00	27.10	0.00	1.00	1.00	1.00	1.00	0.00	1.00	0.00	1.00	0.00	0.00	0.00	0.00	1.00	0.00	0.00	0.00	1.00	1.00	0.00	1.00	2.00	5.00
5607	24	1.00	0.00	0.00	0.00	28.80	0.00	0.00	0.00	0.00	0.00	0.00	0.00	0.00	0.00	0.00	0.00	0.00	0.00	0.00	0.00	0.00	0.00	0.00	0.00	0.00	0.00	0.00	0.00
5610	24	1.00	0.00	0.00	0.00	32.20	0.00	0.00	0.00	0.00	0.00	0.00	0.00	0.00	0.00	0.00	0.00	0.00	0.00	0.00	0.00	0.00	0.00	0.00	0.00	0.00	0.00	0.00	0.00
5614	62	1.00	0.00	0.00	1.00	29.34	0.00	0.00	0.00	1.00	0.00	0.00	1.00	0.00	0.00	0.00	1.00	1.00	0.00	0.00	0.00	0.00	0.00	0.00	1.00	0.00	1.00	2.00	5.00
5617	27	1.00	0.00	0.00	0.00	25.07	1.00	0.00	1.00	1.00	0.00	0.00	0.00	0.00	0.00	0.00	0.00	0.00	0.00	0.00	0.00	0.00	0.00	0.00	0.00	0.00	0.00	0.00	0.00
5622	20	1.00	0.00	0.00	0.00	20.40	0.00	0.00	1.00	0.00	0.00	0.00	0.00	0.00	0.00	0.00	0.00	0.00	0.00	0.00	0.00	0.00	0.00	0.00	0.00	0.00	0.00	1.00	1.00
5625	55	1.00	0.00	0.00	1.00	32.00	0.00	0.00	0.00	1.00	0.00	0.00	0.00	0.00	0.00	0.00	0.00	0.00	0.00	0.00	0.00	0.00	0.00	0.00	0.00	0.00	1.00	0.00	0.00
5631	21	1.00	0.00	0.00	0.00	42.40	0.00	0.00	0.00	0.00	0.00	0.00	0.00	0.00	0.00	0.00	0.00	0.00	0.00	0.00	0.00	0.00	0.00	0.00	0.00	0.00	0.00	1.00	2.00
5636	47	1.00	0.00	0.00	1.00	22.00	0.00	0.00	1.00	0.00	0.00	0.00	0.00	0.00	0.00	0.00	0.00	0.00	0.00	0.00	0.00	0.00	0.00	0.00	0.00	0.00	0.00	0.00	0.00
5644	31	1.00	0.00	0.00	0.00	24.40	0.00	1.00	0.00	0.00	0.00	0.00	0.00	0.00	0.00	0.00	0.00	0.00	0.00	0.00	0.00	0.00	0.00	0.00	0.00	0.00	0.00	1.00	1.00
5652	28	1.00	0.00	0.00	0.00	36.30	0.00	0.00	1.00	1.00	0.00	0.00	0.00	0.00	0.00	0.00	0.00	0.00	0.00	0.00	0.00	0.00	0.00	0.00	0.00	0.00	1.00	0.00	0.00
5653	42	1.00	0.00	1.00	0.00	33.27	0.00	0.00	0.00	0.00	0.00	0.00	0.00	0.00	0.00	0.00	0.00	0.00	0.00	0.00	0.00	0.00	0.00	0.00	0.00	0.00	0.00	1.00	1.00
5657	31	1.00	0.00	0.00	0.00	19.50	0.00	0.00	0.00	0.00	0.00	0.00	0.00	0.00	0.00	0.00	0.00	0.00	0.00	0.00	0.00	0.00	0.00	0.00	0.00	0.00	0.00	0.00	0.00
5660	35	1.00	0.00	0.00	0.00	30.30	0.00	0.00	0.00	1.00	0.00	0.00	0.00	0.00	0.00	0.00	0.00	0.00	0.00	0.00	0.00	0.00	0.00	0.00	0.00	0.00	1.00	2.00	4.00
5665	61	1.00	1.00	0.00	0.00	56.00	0.00	0.00	0.00	0.00	0.00	0.00	0.00	0.00	0.00	0.00	0.00	0.00	0.00	0.00	0.00	0.00	0.00	0.00	0.00	0.00	0.00	1.00	1.00
5671	20	1.00	0.00	0.00	0.00	20.00	0.00	0.00	1.00	0.00	0.00	0.00	0.00	0.00	0.00	0.00	0.00	0.00	0.00	0.00	0.00	0.00	0.00	0.00	0.00	0.00	0.00	1.00	2.00
5674	44	1.00	0.00	1.00	0.00	27.80	0.00	1.00	0.00	0.00	0.00	0.00	0.00	0.00	0.00	0.00	0.00	0.00	0.00	0.00	1.00	0.00	0.00	0.00	0.00	0.00	0.00	1.00	1.00
5677	36	1.00	0.00	0.00	0.00	28.50	1.00	0.00	1.00	0.00	1.00	0.00	0.00	0.00	0.00	0.00	0.00	0.00	0.00	0.00	0.00	0.00	0.00	0.00	0.00	0.00	0.00	0.00	0.00
5681	36	1.00	0.00	0.00	1.00	31.50	0.00	0.00	0.00	0.00	0.00	0.00	0.00	0.00	0.00	0.00	0.00	0.00	0.00	0.00	0.00	0.00	0.00	0.00	0.00	0.00	0.00	1.00	1.00
5683	29	1.00	0.00	0.00	0.00	36.33	0.00	0.00	0.00	0.00	0.00	0.00	0.00	0.00	0.00	0.00	0.00	0.00	0.00	0.00	0.00	0.00	0.00	0.00	0.00	0.00	0.00	1.00	2.00
5686	59	1.00	0.00	0.00	1.00	27.70	1.00	0.00	1.00	0.00	0.00	0.00	0.00	0.00	0.00	0.00	0.00	0.00	0.00	0.00	0.00	0.00	0.00	0.00	0.00	0.00	0.00	1.00	1.00
5689	28	1.00	0.00	0.00	0.00	20.50	0.00	0.00	0.00	0.00	0.00	0.00	0.00	0.00	0.00	0.00	0.00	0.00	0.00	0.00	0.00	1.00	0.00	0.00	0.00	0.00	0.00	1.00	2.00
5691	30	1.00	0.00	0.00	1.00	31.80	0.00	0.00	1.00	0.00	0.00	0.00	0.00	0.00	0.00	0.00	0.00	0.00	0.00	0.00	0.00	0.00	0.00	0.00	0.00	0.00	0.00	1.00	1.00
5692	36	1.00	0.00	0.00	0.00	28.00	0.00	0.00	0.00	1.00	0.00	0.00	0.00	0.00	0.00	0.00	0.00	0.00	0.00	0.00	0.00	0.00	0.00	0.00	0.00	0.00	0.00	0.00	0.00
8022	62	1.00	0.00	1.00	1.00	24.68	0.00	0.00	1.00	0.00	0.00	0.00	0.00	0.00	0.00	0.00	0.00	0.00	0.00	0.00	0.00	0.00	0.00	0.00	0.00	0.00	0.00	0.00	0.00
8026	69	1.00	0.00	0.00	1.00	30.88	1.00	0.00	1.00	0.00	0.00	0.00	0.00	0.00	0.00	0.00	0.00	0.00	1.00	0.00	1.00	0.00	0.00	0.00	1.00	0.00	0.00	1.00	2.00
8029	65	1.00	0.00	0.00	1.00	17.00	1.00	1.00	1.00	0.00	0.00	1.00	0.00	0.00	1.00	0.00	0.00	0.00	0.00	0.00	1.00	1.00	0.00	0.00	1.00	0.00	0.00	1.00	1.00
8100	47	1.00	0.00	0.00	1.00	40.44	0.00	0.00	1.00	0.00	0.00	0.00	0.00	0.00	0.00	0.00	0.00	0.00	1.00	0.00	0.00	0.00	0.00	0.00	1.00	0.00	0.00	1.00	2.00
8107	58	1.00	0.00	0.00	1.00	44.77	0.00	0.00	0.00	0.00	0.00	0.00	0.00	0.00	0.00	0.00	0.00	0.00	0.00	0.00	0.00	0.00	0.00	0.00	0.00	0.00	0.00	0.00	0.00
8331	32	1.00	0.00	0.00	1.00	27.18	0.00	0.00	1.00	0.00	0.00	0.00	0.00	0.00	0.00	0.00	0.00	0.00	0.00	0.00	0.00	0.00	0.00	0.00	0.00	0.00	0.00	0.00	0.00
8964	23	0.00	0.00	0.00	1.00	20.12	0.00	0.00	1.00	0.00	0.00	0.00	0.00	1.00	0.00	0.00	0.00	0.00	0.00	0.00	0.00	0.00	0.00	0.00	0.00	0.00	1.00	1.00	1.00

n/a = not available
