# Supplementary material for: Apolipoprotein L1 risk variants associate with prevalent atherosclerotic disease in African American systemic lupus erythematosus patients
Source: PLoS One. 2017 Aug 29;12(8):e0182483. doi: 10.1371/journal.pone.0182483 (PMC5574561; doi:10.1371/journal.pone.0182483)
Supplement: S2 Table — (PDF) [file pone.0182483.s002.pdf]

S2 Table. Definitions of data bank abbreviations used in S1 Table.

ABBREVIATION	DEFINITION
D1_PT_ID	Patient ID
D1_Age	Age in years
D1_Sex	Gender (1.00 = female; .00 = male)
D2_DM	Diabetes Mellitus
D2_smoking	History of smoking current or past
D2_HTN	Hypertension
D2_BMI	Body Mass Index
D2_Statin	Current statin prescription
D3_AVN	Avascular Necrosis
D3_Nephritis	History of lupus nephritis
D3_APS	Antiphospholipid syndrome
D3_ESRD	End stage renal disease
D4_cardiac_arrest	History of cardiac arrest
D4_arrythmia	History of arrhythmia
D4_LVH	Left ventricular hypertrophy on echocardiogram
D4_CHF	History of congestive heart failure
D5_AAA	History of abdominal aortic aneurysm
D5_Stroke	History of transient ischemic attack or Stroke
D5_CEA	Past carotid artery repair
D5_carotid_stenosis	History of carotid stenosis
D5_Posit_Stress	Past positive stress test
D5_angina	History of angina
D5_MI	Past Myocardial Infarction
D5_CA_Revasc	Past coronary artery revascularization
D5_CA_Calc	Coronary artery calcifications on imaging
D5_Vasc_Calc	Vascular calcifications on imaging
D5_PVD	Peripheral vascular disease
D6_TE	History of pulmonary embolism or DVT
D8_number_risk_alleles	Number of APOL1 risk alleles
D8_APOL1_status	APOL1 genotype
